# The complete mitochondrial genome of *Somanniathelphusa boyangensis* and phylogenetic analysis of Genus *Somanniathelphusa* (Crustacea: Decapoda: Parathelphusidae)

**DOI:** 10.1371/journal.pone.0192601

**Published:** 2018-02-13

**Authors:** Xin-nan Jia, Shu-xin Xu, Jun Bai, Yi-fan Wang, Zong-heng Nie, Chun-chao Zhu, Yan Wang, Yi-xiong Cai, Jie-xin Zou, Xian-min Zhou

**Affiliations:** 1 Research lab of Freshwater Crustacean Decapoda & Paragonimus, School of Basic Medical Sciences, Nanchang University, Nanchang, Jiangxi, PR China; 2 Institute of Pathogen Biology, Jiangxi Academy of Medical Sciences, Nanchang, Jiangxi, PR China; 3 Key Laboratory of Poyang Lake Environment and Resource Utilization, Ministry of Education, Nanchang University, Nanchang, Jiangxi, PR China; 4 National Biodiversity Centre, National Parks Board, Singapore, Singapore; National Cheng Kung University, TAIWAN

## Abstract

In this study, the authors first obtained the mitochondrial genome of *Somanniathelphusa boyangensis*. The results showed that the mitochondrial genome is 17,032bp in length, included 13 protein-coding genes, 2 rRNAs genes, 22 tRNAs genes and 1 putative control region, and it has the characteristics of the metazoan mitochondrial genome A+T bias. All tRNA genes display the typical clover-leaf secondary structure except *tRNA*^*Ser(AGN)*^, which has lost the dihydroxyuridine arm. The GenBank database contains the mitochondrial genomes of representatives of approximately 22 families of Brachyura, comprising 56 species, including 4 species of freshwater crab. The authors established the phylogenetic relationships using the maximum likelihood and Bayesian inference methods. The phylogenetic relationship indicated that the molecular taxonomy of *S*. *boyangensis* is consistent with current morphological classification, and Parathelphusidae and Potamidae are derived within the freshwater clade or as part of it. In addition, the authors used the *COX1* sequence of *Somanniathelphusa* in GenBank and the *COX1* sequence of *S*. *boyangensis* to estimated the divergence time of this genus. The result displayed that the divergence time of *Somanniathelphusa qiongshanensis* is consistent with the separation of Hainan Island from mainland China in the Beibu Gulf, and the divergence time for *Somanniathelphusa taiwanensis* and *Somanniathelphusa amoyensis* is consistent with the separation of Taiwan Province from Mainland China at Fujian Province. These data indicate that geologic events influenced speciation of the genus *Somanniathelphusa*.

## Introduction

Brachyura within Decapoda is a relatively large group within Crustacea and includes 1,271 genera and approximately 7,000 species worldwide. Freshwater crabs differ from the other members of Brachyura, and the taxonomy of freshwater crabs is divided into three superfamilies: Pseudothelphusoidea (distributed in America), Gecarcinucoidea (distributed in Asia, Australia and Africa) and Potamoidea (distributed in Asia, Europe, and Africa)[1]. Approximately 2,000 species of freshwater crabs are included in these 3 superfamilies worldwide, and new species of freshwater crabs are still being described[1]. Freshwater crabs of the Potamoidea and Gecarcinucoidea are found in mainland China. The Potamoidea includes 34 genera in China, while Gecarcinucoidea includes only a single genus (*Somanniathelphusa*) in China[2]. The Chinese mainland has a vast territory and provides a variety of environments for freshwater crabs, resulting in diverse genera and species. Compared with Potamoidea, Gecarcinucoidea includes 25 species of the genus *Somanniathelphusa* in mainland China[2]. *Somanniathelphusa* species are mainly found in low-altitude rivers, lakes, ditches, and mud holes in paddy fields and grass, whereas Potamoid crabs occur in the streams of mountainous and hilly regions. Some species of *Somanniathelphusa* exist as second intermediate hosts of flukes of the genus Paragonimus[2]. Sequencing the mitochondrial genome and performing phylogenetic analyses of *Somanniathelphusa* are important tasks.

Brachyura belongs to Arthropoda, Crustacea, Malacostraca and Decapoda, and the associated species are considered true crabs. Although most species of Brachyura are distributed in oceans, the changes that occurred over the earth’s geological history produced gradual adaptations to freshwater life in Brachyura. The entire life cycle of the unique group of freshwater crabs now develops in freshwater ecosystems[2]. A comparison of the ontogenesis of freshwater crabs with that of other marine crab groups shows that the mating system and morphological characteristics of the eggs are different. The GenBank database contains the mitochondrial genome of approximately 22 families of Brachyura, including 56 species, and the mitochondrial genomes of 4 species of freshwater crab: *Potamiscus motuoensis*, *Geothelphusa dehaani*[3], *Huananpotamon lichuanense*[4] and *Sinopotamon xiushuiense*[5]. *Potamiscus motuoensis* (GenBank accession no. KY285013), *H*. *lichuanense* (GenBank accession no. KX639824) and *S*. *xiushuiense* (GenBank accession no. KU042041) were submitted by our laboratory. GenBank does not contain data on the freshwater crab genus *Somanniathelphusa*. The species *Somanniathelphusa boyangensis* was first described in 1994[6], and we submitted the mitochondrial genome of *S*. *boyangensis* to GenBank in November 2016 (GenBank accession no. KU042042).

Based on the genetic distance between organisms, the molecular evolution rate can be established and used to determine the origin or divergence time of different taxa. This indirect time data can be compared with direct data obtained from the fossil record. Therefore, molecular clock research provides a new method of exploring early evolution and verifying conclusions based on the fossil record. Moreover, molecular clock research is also of special significance for determining the origins of biological groups with incomplete fossil records[7]. Divergence time estimates are the foundation of molecular clock research, and they have been widely applied to analyze protozoa and metazoa[8–14]. Tsang et al. studied the divergence time of Brachyura[15] and suggested that Brachyura diverged at 212.91 Ma (million years from the present) and freshwater crabs diverged at 136.96 Ma. However, divergence time estimates of the freshwater crab genus *Somanniathelphusa* have not been reported; thus, we estimated the divergence time for this genus.

## Materials and methods

### Ethics statement

There are no specific permits required for crab collection in the study locations. The sampling locations are not privately owned or natural protected areas. The crab species used in this study are not considered endangered or protected, and their collection is legal in China.

### Sequencing and assembling the mitochondrial genome

In this study, the author Xianmin Zhou collected *Somanniathelphusa boyangensis* (catalogue number JX20140927) specimens at the village of Hongwei in the town of Nanjishan and at Poyang Lake (28°56'53.7"N 116°21'47.1"E) in Jiangxi Province, China, in 2014. The specimens were preserved in 100% ethanol and then stored at 4°C until DNA extraction. Total DNA was extracted from the leg muscle tissue of individual crabs using the Aidlab Genomic DNA Extraction Kit (Aidlab, China) according to the manufacturer’s instructions. Primers designed to match generally conserved regions of Potamidae mitochondrial DNA were used to amplify short fragments of the *16S*, *12S*, *COX1*, *ATP6*, *COX3*, *ND4*, *Cyt b*, and *ND1* genes. Specific primers listed in S1 Table were designed based on sequences in these conserved regions and used to amplify mtDNA sequences via several PCR reactions. The PCR reactions were performed using 5 μL of 10 × LA Taq Buffer II (Mg2+Plus), 8 μL of dNTP mixture, 2 μL of each of the primers, 2μL of DNA template, 0.5 μL TaKaRa LA Taq polymerase (5 U/μL) and ddH2O to a final volume of 50 μL. The PCR reactions were conducted with LA Taq polymerase under the following conditions: 35 cycles of denaturation at 94°C for 30 seconds, annealing with the primers at 50°C for 30 seconds, and then an extension at 72°C for 1 minute per 1 kilobase. The PCR products were cloned into a pGEM-T vector (Promega, USA) and then sequenced or sequenced directly using the dideoxynucleotide procedure and an ABI 3730 automatic sequencer. The sequences were assembled using DNAStar software and adjusted manually to generate the complete mitochondrial DNA sequence.

### Mitochondrial genome annotation

Most tRNA annotations were performed using the web server tRNA-scan SE 1.21[16]. Other tRNAs and rRNAs were identified via BLAST searches and comparisons of the results with those of similar Brachyura, such as *Sinopotamon xiushuiense*[5] and *Geothelphusa dehaani*[3]. These sequence data were submitted to the NCBI database. Based on the positions of the tRNAs and rRNAs, the approximate locations of 13 protein-coding genes (PCGs) were calculated, and PCG annotations were performed using SeqBuilder software (DNAStar)[17]. The secondary structure of the tRNA genes was putatively predicted using the web server ARWEN[18]. Gene maps of the mitochondrial genome were constructed using the web server OGDraw v1.1 (http://ogdraw.mpimp-golm.mpg.de).

### Phylogenetic analysis

Because of the lack of mitochondrial genome data for freshwater crabs and the *ND1* and *ND2* PCGs of *Sinopotamon yangtsekiens*e, a phylogenetic analysis was performed using 11 PCG sequence data for Brachyura from GenBank together with our sequence data of the mitochondrial genome of *Somanniathelphusa boyangensis*. Only five mitochondrial genomes of freshwater crabs have been previously reported and submitted to GenBank: *Geothelphusa dehaani*[3], *Sinopotamon xiushuiense*[5], *Huananpotamon lichuanense*[4], *Potamiscus motuoensis* and *S*. *yangtsekiense* (not complete). In total, 59 species covering all major Brachyura groups were used in the analyses, and *Panulirus ornatus* (GenBank number no. GQ223286) was chosen as the outgroup (S2 Table). MAFFT software[19] was used to compare the sequences, and the Gblocks Server (http://molevol.cmima.csic.es/castresana/Gblocks_server.html) was used to select the conserved sequence area. The best fitting evolutionary model for the maximum likelihood (ML) analyses of the 11 PCGs was GTR+G+I, and it was selected using ModelGenerator[20]. This model was used in MEGA 6.0[21], and 1,000 bootstrap replicates were performed. For the Bayesian inference (BI) analyses, the best fitting evolutionary model (GTR+G+I) was determined using MrModeltest 2.3[22]. The BI analyses were performed with MrBayes 3.2.6[23], which used Markov Chain Monte Carlo (MCMC) with four chains to run 2 million generations. Sampling was performed every 1,000 generations, and the initial 500,000 steps were discarded as burn-in. The consensus BI trees were visualized using FigTree 1.4.0 software.

### Divergence time estimation

To estimate the divergence time of the genus *Somanniathelphusa* using GenBank data, we chose 14 complete *COX1* mitochondrial gene sequences (S3 Table) of the genus *Somanniathelphusa* to correct the fossil time (Table 1)[24–28]. We used the strict clock of BEAST v1.8.2[29] software to estimate the divergence time of each clade based on the sequence of *COX1* and selected a normal prior distribution. The best fitting evolutionary model was GTR+G+I using ModelGenerator[20] software, and the Yule process was used as the tree prior shared and random starting tree. In total, 200,000,000 generations were run, and the parameters were logged every 20,000 generations, with the first 10% discarded as burn-in. TreeAnnotator v1.8.2 software was used to generate the tree, and the divergence time was visualized using FigTree 1.4.0 software.

**Table 1:** Fossil calibrations used in divergence time estimation.

Classification	Species or genus with available mitochondrial genome data	Age
Family Portunidae	genus *Portunus*, genus *Callinects*	33.9–56 Ma^a^
Family Portunidae	genus *Scylla*	33.9–37 Ma
Family Portunidae	genus *Charybdis*	33.9–35 Ma
Subsection Raninoida	*Umalia orientalis*, *Lyreidus brevifrons*, *Ranina ranina*	130.8–133.9 Ma
Family Homolidae	*Homologenus malayensis*, *Homola orientalis*, *Moloha majora*	145–152.1 Ma

^a^ Ma, million years for the present

## Results and discussion

### Genome structure and organization

**Fig 1:**
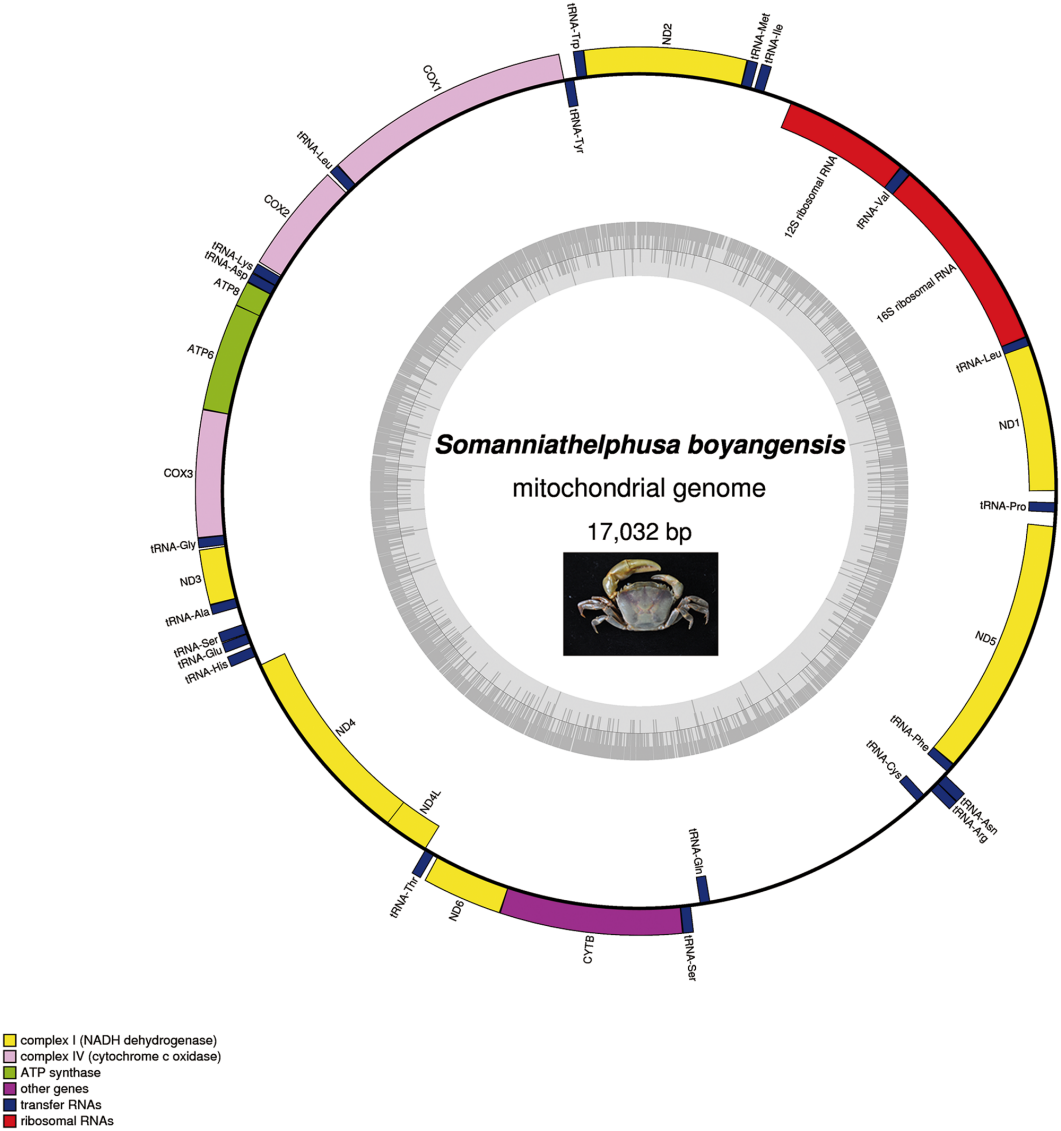
Gene map of *Somanniathelphusa boyangensis* mitochondrial genome.

The mitochondrial genome of *Somanniathelphusa boyangensis* was 17,032 bp in length and contained 37 genes (2 rRNAS, 22 tRNAs and 13 PCGs) with homologues in other metazoan mitochondrial genomes. These genes showed a strong A+T bias (S4 Table). According to the mitochondrial genome data of Brachyura published in GenBank, most of the mitochondrial genome of the Brachyura is 15–16 kb, including the freshwater crab *Huananpotamon lichuanense*[4] (15,380 bp). *Sinopotamon xiushuiense* (18,460 bp), *Geothelphusa dehaani* (18,197 bp) and *Potamiscus motuoensis* (17,971 bp) are freshwater crabs with mitochondrial genomes longer than 17 kb. In the mitochondrial genome of *S*. *boyangensis*, the H-strand encodes 24 genes and the L-strand encodes 13 genes. The coding regions of the *S*. *boyangensis* genome totaled 14,746 bp in length, and the non-coding regions totaled 2,313 bp. The mitochondrial genome data of freshwater crabs published in GenBank show that their coding regions are approximately 14,800 bp in length and the non-sequences gene varies greatly in length; therefore, the length of the mitochondrial genome is mainly determined by the length of the non-coding regions[30]. Gene overlaps and non-coding regions are frequently found in decapod mitochondrial genomes[31–35], and this was also the case with the freshwater crabs. The mitochondrial genome of *S*. *boyangensis* had 19 non-coding regions, which were between 1 and 1,516 bp in length, and the longest was the 1,516 bp region between *tRNA*^*Gln*^ and *tRNA*^*Cys*^. Eight gene overlaps of 1–10 bp in length were observed, and the longest was a 10 bp overlap between *ND1* and *tRNA*^*Leu(CUN)*^. The gene map of *S*. *boyangensis* is shown in Fig 1.

### Protein-coding genes and codon usage

The mitochondrial genome consisted of 13 PCGs, which is similar to that of other Brachyura. Nine PCGs were encoded by the H-strand (*COX1*, *COX2*, *COX3*, *ATP6*, *ATP8*, *ND2*, *ND3*, *ND6* and *Cyt b*), and four were encoded by the L-strand (*ND1*, *ND4*, *ND4L* and *ND5*). Four reading-frame overlaps were observed on the same strand as observed in other Brachyura (*ATP8* and *ATP6* shared four nucleotides; *ATP6* and *COX3* shared one nucleotide; *ND4* and *ND4L* shared seven nucleotides; and *ND6* and *Cyt b* shared one nucleotide). Eight PCGs used ATG initiator codons (*COX1*, *COX2*, *COX3*, *ATP8*, *Cyt b*, *ND2*, *ND4*, and *ND4L*), and the other PCGs used ATA as an initiator codon. The most frequent termination codons were TAA (*COX1*, *COX2*, A*TP6*, *ATP8*, *ND1*, *ND6*, and *ND4L*) and TAG (*ND2*, *ND3*, and *ND4*); and the termination codons of *COX3*, *ND5*, and *Cyt b* were abbreviated T (Table 2). The A+T content of the PCGs was 69.8%, with the highest A+T content observed in *ATP8* (76.7%) and the lowest observed in *COX3* (63.8%). According to the GenBank data, the initiation codons of the mitochondrial genome PCGs of Brachyura are relatively conserved. Most initiation codons are ATN, although uncommon initiation codons were found, including GTG and ACG. The termination codons mainly contained TAA, TA and T, and TAG was rare. Special initiation codons could be converted to a common codon by an RNA editing function[36], which allowed translation to proceed normally. Incomplete initiation codons could be converted to complete ones by the polyadenylation function after transcription[37].

**Table 2:** Characteristics of genes in the mitochondrial genome of *Somanniathelphusa boyangensis*.

Gene	Position		Size (bp)	Condon			Intergenic nucleotide (bp)^b^	Strand^c^
	From	To		Amino acid	Start	Stop^a^		
*COX1*	1	1536	1536	511	ATG	TAA	0	H
*tRNA*^*Leu(UUR)*^	1537	1599	63				0	H
*COX2*	1617	2300	684	227	ATG	TAA	17	H
*tRNA*^*Lys*^	2311	2375	65				10	H
*tRNA*^*Asp*^	2377	2439	63				1	H
*ATP8*	2440	2598	159	52	ATG	TAA	0	H
*ATP6*	2595	3266	672	223	ATA	TAA	-4	H
*COX3*	3266	4055	790	263	ATG	T--	-1	H
*tRNA*^*Gly*^	4056	4117	62				0	H
*ND3*	4127	4471	345	114	ATA	TAG	9	H
*tRNA*^*Ala*^	4471	4532	62				-1	H
*tRNA*^*Ser(AGN)*^	4646	4711	66				113	H
*tRNA*^*Glu*^	4714	4777	64				2	H
*tRNA*^*His*^	4804	4870	67				26	H
*ND4*	4932	6269	1338	445	ATG	TAG	61	L
*ND4L*	6263	6565	303	100	ATG	TAA	-7	L
*tRNA*^*Thr*^	6568	6630	63				2	H
*ND6*	6659	7159	501	166	ATA	TAA	28	H
*Cyt b*	7159	8293	1135	378	ATG	T--	-1	H
*tRNA*^*Ser(UCN)*^	8294	8360	67				0	H
*tRNA*^*Gln*^	8423	8490	68				62	L
*tRNA*^*Cys*^	10007	10071	65				1516	L
*tRNA*^*Arg*^	10121	10186	66				49	H
*tRNA*^*Asn*^	10186	10256	71				-1	H
*tRNA*^*Phe*^	10282	10344	63				25	L
*ND5*	10345	12055	1711	570	ATA	T--	0	L
*tRNA*^*Pro*^	12145	12210	66				89	L
*ND1*	12285	13256	972	323	ATA	TAA	74	L
*tRNA*^*Leu(CUN)*^	13247	13311	65				-10	L
*16S rRNA*	13312	14625	1314				0	L
*tRNA*^*Val*^	14626	14698	73				0	L
*12S rRNA*	14699	15532	834				0	L
*Putative control region*	15533	15720	188				0	
*tRNA*^*Ile*^	15721	15785	65				0	H
*tRNA*^*Met*^	15808	15873	66				22	H
*ND2*	15874	16884	1011	336	ATG	TAG	0	H
*tRNA*^*Trp*^	16883	16948	66				-2	H
*tRNA*^*Tyr*^	16968	17032	65				19	L

^a^ T--represents incomplete stop codons

^b^ Numbers correspond to the nucleotides separating adjacent genes. Negative numbers indicate overlapping nucleotides.

^c^ H and L indicate that the gene is encoded by the H- and L-strand, respectively.

### Transfer RNA genes

**Fig 2:**
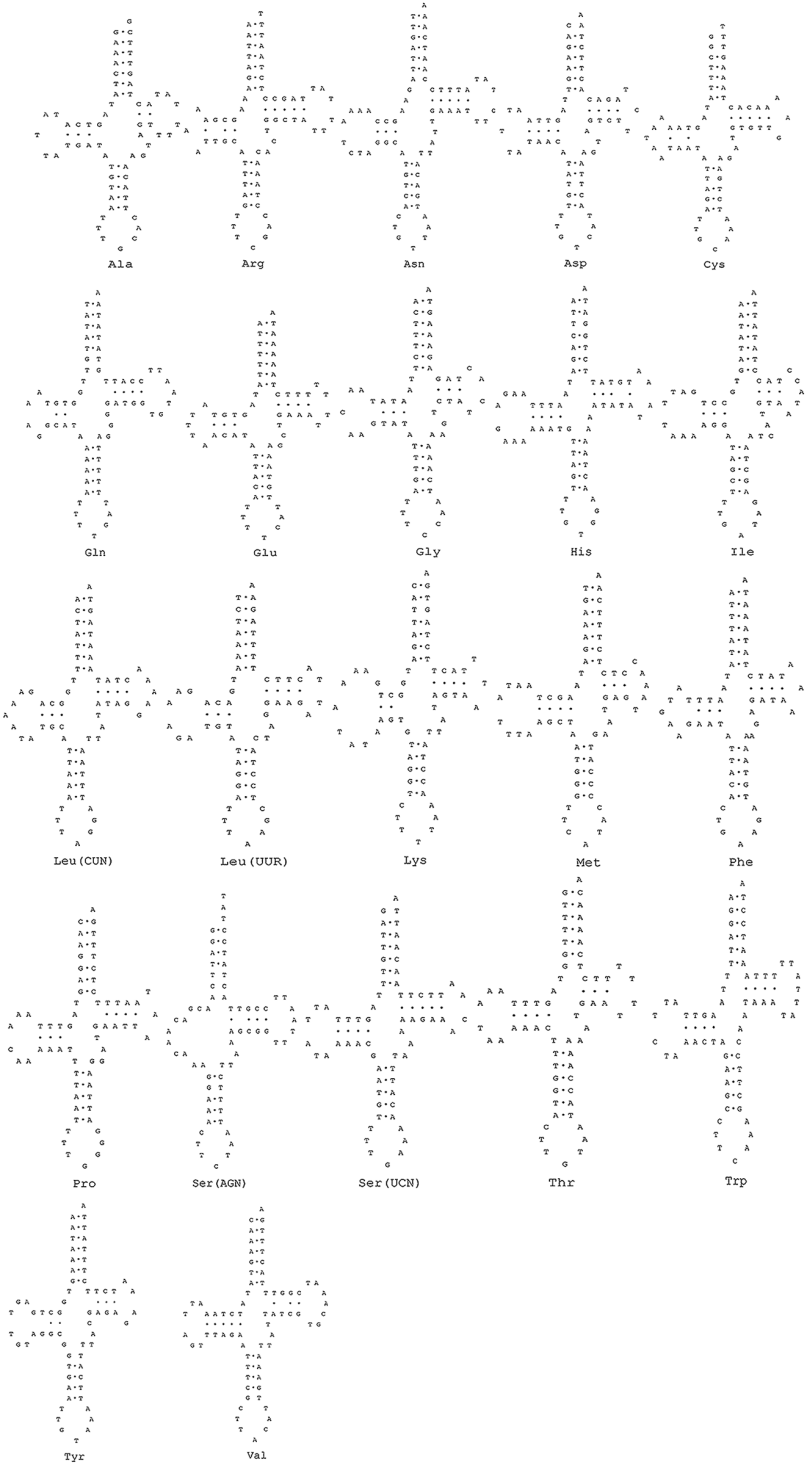
Putative secondary structures of 22 tRNAs encoded by the mitochondrial genome of *Somanniathelphusa boyangensis*.

All tRNA genes display the typical clover-leaf secondary structure except *tRNA*^*Ser(AGN)*^, which has lost the dihydroxyuridine arm (DHU arm), a feature that is commonly observed in metazoan mitochondrial genomes (Fig 2)[38]. The tRNA genes range in size from 62 (*tRNA*^*Ala*^) to 73 (*tRNA*^*Val*^) bps. Fifteen tRNA genes were encoded by the H-strand, whereas the remainder were encoded by the L-strand. The 21 tRNA (except *tRNA*^*Trp*^) genes possessed a common length of 7 bp for the aminoacyl stem, whereas the aminoacyl stem of the *tRNA*^*Trp*^ gene was 6 bp. All tRNAs were found to have the DHU arm (except *tRNA*^*Ser(AGN)*^), the TψC arm and the anticodon arm. The tRNA genes of *Somanniathelphusa boyangensis* contained 39 unmatched base pairs, including thirty G-T mismatches, three T-T mismatches, two A-A mismatches, two A-C mismatches, one C-C mismatch and one A-G mismatch. In most Brachyura mitochondrial genomes, the length of the tRNA was 60–80 bp, and all show the typical clover-leaf structure. Furthermore, the length of the aminoacyl stem, the DHU arm, the anticodon arm and the TΨC arm were 7 bp, 4 bp, 5 bp and 5 bp, respectively. Many species of Brachyura lacked the DHU arm of tRNA^*Ser(AGN)*^, which is widely found in metazoan mitochondrial genomes[30, 31, 38, 39]. This particular tRNA secondary structure has also been found in mammals and insects[40, 41]. In Brachyura, special anticodons of mitochondrial tRNAs were also common, such as the anticodons of tRNA^*Lys*^ and tRNA^*Ser(AGN)*^, which were TTT and TCT, although they are normally CTT and GCT. Although the third codon swing phenomenon of tRNAs is not uncommon, tRNA anticodons are usually more conservative in invertebrates. The tRNAs also presented the unmatched base pairs phenomenon; however, mismatches in these tRNAs are modified by RNA editing[42].

### Ribosomal RNA genes

Similar to the *16S rRNA* and *12S rRNA* genes in the mitochondrial genome of other Brachyura species, the *16S rRNA* and *12S rRNA* genes of *Somanniathelphusa boyangensis* were encoded by the L-strand. The length of the *16S rRNA* and *12S rRNA* genes was 1,314 bp and 834 bp, respectively. The *16S rRNA* and *12S rRNA* genes were located on the L-strand between the *tRNA*^*Leu*^ gene and the *tRNA*^*Val*^ gene and between the *tRNA*^*Val*^ gene and the putative control region, respectively, which is typical for pancrustaceans.

### Non-coding regions and the putative control region

*Somanniathelphusa boyangensis* had 19 non-coding regions. The putative control region was identified between the *12S rRNA* and the *tRNA*^*Ile*^ gene, and it had a length of 188 bp (Table 2). According to the published mitochondrial genome data of Brachyura, the length of the control region is between 500 and 1,500 bp. The location of the control region of the *S*. *boyangensis* mitochondrial genome putative is typical for pancrustaceans, and it had a much higher A+T content (87.8%; A = 43.1%, G = 1.6%, T = 44.7%, C = 10.6%) than the overall genome (72.3%). However, the region was only 188 bp in length, which is inconsistent with the published control regions of Brachyura mitochondrial genomes. Based on the large length difference, we inferred that this sequence was the putative control region. The other 18 non-coding regions varied from 1 to 1,516 bp in length. The length of the non-coding region close to the control region was 1,516 bp, which was between tRNA^*Cys*^ and tRNA^*Gln*^. The A+T content of the non-coding region was 78.1%, which was lower than that of the putative control region; therefore, we suggest that the non-coding region was not a control region. The length of the putative control region of different species was generally derived from a highly repetitive sequence, and this sequence could further form a stem loop from a simple hairpin structure to a complex secondary structure[43]. The mitochondrial genome putative control region of crustaceans contains conserved sequences, such as an AT-box and a poly-T sequence, which may be associated with mitochondrial DNA replication[44].

### Phylogenetic relationships

**Fig 3:**
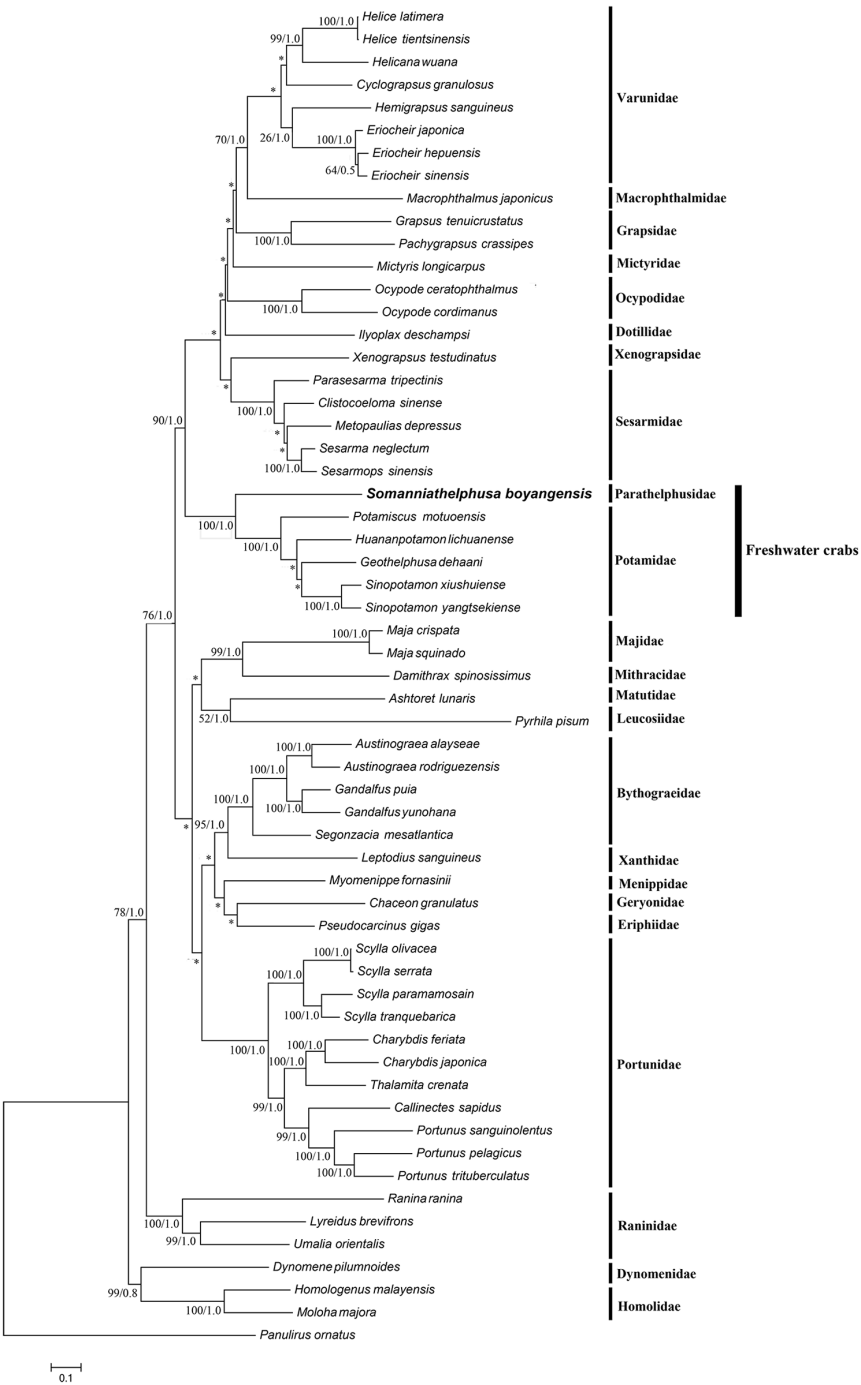
Phylogenetic maximum likelihood (ML) tree of *Somanniathelphusa boyangensis* and related brachyurans based on 11 PCGs sequences from the mitochondrial genome; *Panulirus ornatus* serves as the outgroup. The numbers at the internodes are maximum likelihood (ML) bootstrap proportions and Bayesian inference (BI) posterior proportions. The differences between the ML and BI trees are indicated by ‘*’. The scale bars represent genetic distance.

We used the 11 PCGs of the mitochondrial genomes of Brachyura published in GenBank for *Somanniathelphusa boyangensis* and *Panulirus ornatus* (outgroup) to establish the phylogenetic relationships using the ML and BI methods. The phylogenetic relationship results for the ML and BI show a highly similar topology structure, which presents high bootstrap support (Fig 3). The phylogenetic relationship indicated that the molecular taxonomy of *S*. *boyangensis* is consistent with the morphological classification, and *S*. *boyangensis* of Parathelphusidae and the 5 species of Potamidae (*Geothelphusa dehaani*, *Sinopotamon yangtsekiense*, *Huananpotamon lichuanense*[4], *Potamiscus motuoensis* and *Sinopotamon xiushuiense*) are derived within the freshwater crab clade or as part of it. According to this phylogenetic relationship, the molecular classification of the mitochondrial genome PCGs can be used to classify Brachyura, and Parathelphusidae (Gecarcinucoidea) and Potamidae (Potamoidea) are derived within the freshwater crab clade or as part of it. Thus, these results indicate that freshwater crabs have a close evolutionary relationship. Because of the lack of mitochondrial genome data for certain species, the results of the ML and BI analyses are different, which shows the effect of long-branch attraction (LBA). This finding suggests that data accumulation is important for establishing system generation processes.

### Divergence time estimation for the genus *Somanniathelphusa*

**Fig 4:**
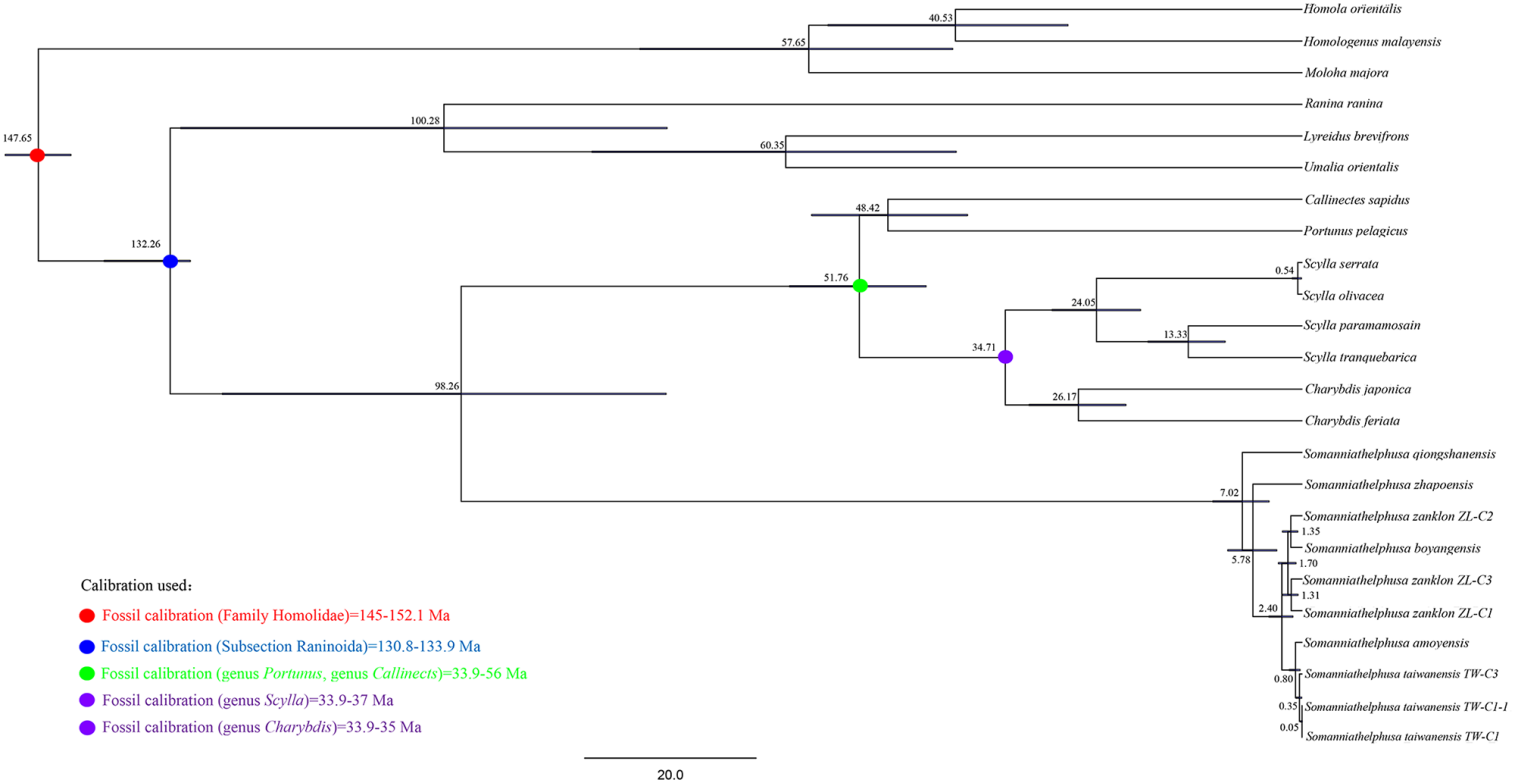
Divergence time of Genus *Somanniathelphusa* base on *COX1* sequence of the mitochondrial genome. The scale bars represent millions of years from the present.

We used the *COX1* sequence of *Somanniathelphusa* in GenBank and the *COX1* sequence of *Somanniathelphusa boyangensis* to estimate the divergence time of this genus. The results showed that the estimated divergence time for *Somanniathelphusa qiongshanensis* (distributed in Qiongshan, Hainan)[2] was approximately 7.02 Ma (million years from the present) (95% credibility interval = 3.89–10.47 Ma); the estimated divergence time for *Somanniathelphusa zhapoensis* (collected in Zhapo, Guangdong)[45] was approximately 5.78 Ma (95% credibility interval = 2.99–8.73 Ma); and the estimated divergence time for *Somanniathelphusa amoyensis* (distributed in Xiamen and Fuzhou, Fujian)[2] and *Somanniathelphusa taiwanensis* (distributed in Jiayi, Taiwan)[2] was approximately 0.80 Ma (95% credibility interval = 0.27–1.53 Ma). However, *S*. *boyangensis* did not present good separation from *Somanniathelphusa zanklon* (*Somanniathelphusa zanklon ZL-C1* collected in Dongguan, Guangdong; *Somanniathelphusa zanklon ZL-C2* collected in Nanhai, Guangdong; and *Somanniathelphusa zanklon ZL-C3* collected in Su Kwun, Hong Kong)[45] (Fig 4). In this study, we used molecular clock research to estimate the divergence time for the genus *Somanniathelphusa*, and the results show that the estimated divergence time was consistent with the related data. Geological data indicate that the Indian Plate and Eurasian Plate collided approximately 50 Ma ago, and after the collision, the smaller Asian continent had decreased in size by nearly 1,500 km, and the crust of the Qinghai-Tibet Plateau was approximately twice as thick as the normal continental crust (35–40 km). The direction of crustal movement was to the east (move toward the Pacific) and caused by differential stress, which caused the southeast Asian Plate (Western Yunnan China, Vietnam, Laos, Cambodia, and Thailand) to move to the southeast and spin to the right before 24 Ma. The differential stress also caused Hainan Island to rotate to its present position. The Hainan island began to separate from the Beibu Gulf of mainland China gradually in 24 Ma and continues to separate[46]. We calculated that the divergence time of *S*. *qiongshanensis* (distributed in Qiongshan, Hainan) was 7.02 Ma (95% credibility interval = 3.89–10.47 Ma), which is consistent with the gradual separation of Hainan Island from mainland China in China's Beibu Gulf. In addition, the geology data also indicate that the Early Pleistocene, Middle Pleistocene, Late Pleistocene and Holocene periods of the Quaternary experienced glacial and interglacial periods. The decline of the sea surface during these glacial periods connected Taiwan Province with Fujian Province, and the snowmelt and sea surface increased during the interglacial periods, which caused Taiwan Province to separate from Fujian Province. The four glacial and interglacial periods (1.50–1.10 Ma, 0.90–0.40 Ma, 0.20–0.11 Ma, and 0.01–0.00 Ma) led to connections and separations between Taiwan Province and Fujian Province[47]. We calculated that the divergence time of *S*. *amoyensis* (distributed in Xiamen and Fuzhou, Fujian) and *S*. *taiwanensis* (distributed in Jiayi, Taiwan) was at approximately 0.80 Ma (95% credibility interval = 0.27–1.53 Ma). This result is consistent with the four glacial and interglacial periods of the Quaternary period and the estimated time at which genus *Nanhaipotamon* reached Taiwan[48].

However, *Somanniathelphusa boyangensis* did not separate fully from *Somanniathelphusa zanklon*. We speculate that the freshwater crab genus *Somanniathelphusa* (Parathelphusidae) is one of the most adaptable relative to the other 34 genera of Potamidae in China. This genus is environmentally adaptable and inhabits streams, rivers and lakes, predominantly rivers and lakes. The other species of freshwater crabs had narrower distributions and ecological limitations compared with *Somanniathelphusa*. Therefore, the dispersal of *Somanniathelphusa* was more "convenient" compared with that of the other 34 genera of freshwater crabs. According to our results, the divergence time of *Somanniathelphusa qiongshanensis* is consistent with the separation of Hainan Island from mainland China in Beibu Gulf, and the divergence time for *Somanniathelphusa taiwanensis* and *Somanniathelphusa amoyensis* is consistent with the separation of Taiwan Province from Mainland China at Fujian Province. These findings indicate that geologic events influenced speciation in the genus *Somanniathelphusa*.

### Gene rearrangement

**Fig 5:**
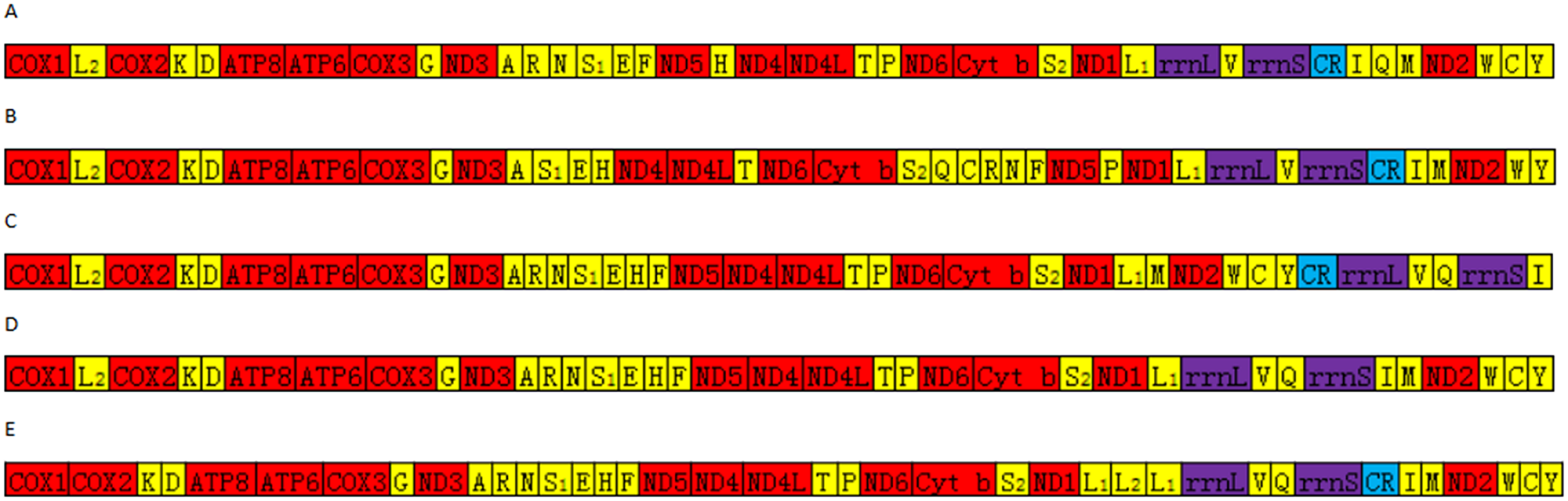
Gene rearrangements of freshwater crabs. A represents the original gene order of Pancrustacea; B represents *Somanniathelphusa boyangensis*; C represents *Sinopotamon xiushuiense*; D represents *Huananpotamon lichuanense*; E represents *Geothelphusa dehaani*.

Crustaceans have a highly rearranged mitochondrial structure, and tRNA gene rearrangements are the most common, whereas PCGs and rRNA genes are less rearranged. Genome rearrangement was determined using the original gene order of Pancrustacea as a reference. According to the current published mitochondrial genome of Potamoidea (except *Potamiscus motuoensis*), a *tRNA*^*His*^ shift (a shift between the *ND5* and *ND4* genes was rearranged to between *tRNA*^*Glu*^ and *tRNA*^*Phe*^) was observed in the freshwater crabs *Sinopotamon xiushuiense*, *Huananpotamon lichuanense*[4] and *Geothelphusa dehaani*, and the *tRNA*^*Gln*^ of these three freshwater crabs was rearranged from between *tRNA*^*Ile*^ and *tRNA*^*Met*^ to between *tRNA*^*Val*^ and *12S rRNA* (Fig 5). *Somanniathelphusa boyangensis* did not exhibit this gene rearrangement, although a rare PCG rearrangement was observed in which the adjacent genes *tRNA*^*Arg*^ and *tRNA*^*Asn*^, PCG *ND5* and its adjacent *tRNA*^*Phe*^, *tRNA*^*Gln*^, *tRNA*^*Cys*^ and *tRNA*^*Pro*^ formed the gene block of *tRNA*^*Gln*^-*tRNA*^*Cys*^-*tRNA*^*Arg*^-*tRNA*^*Asn*^-*ND5*-*tRNA*^*Phe*^-*tRNA*^*Pro*^, and then the gene block was transferred to between *tRNA*^*Ser(UCN)*^ and PCG *ND1*. According to data released by the NCBI, the mitochondrial genomes of freshwater crabs are not well sequenced. One of the three superfamilies of freshwater crabs, Gecarcinucoidea, was only represented by the mitochondrial genome of *S*. *boyangensis*, which was obtained by our laboratory, and the monophyly of Gecarcinucoidea was not supported based on the gene rearrangement results.

## Conclusion

In this study, the authors first obtained the mitochondrial genome of *Somanniathelphusa boyangensis*. The mitochondrial genome was 17,032 bp in length and contained 13 protein-coding genes, 2 rRNAs genes, 22 tRNAs genes and 1 putative control region. The phylogenetic analyses indicated that the molecular taxonomy of *S*. *boyangensis* is consistent with the morphological classification. Furthermore, they indicated that *S*. *boyangensis* of Parathelphusidae and 5 species of Potamidae (*Geothelphusa dehaani*, *Sinopotamon yangtsekiense*, *Huananpotamon lichuanense*, *Potamiscus motuoensis* and *Sinopotamon xiushuiense*) are derived within the freshwater crab clade or as part of it. These results indicate that freshwater crabs have close evolutionary relationships. The estimated divergence time for *Somanniathelphusa qiongshanensis* is approximately 7.02 Ma, and the estimated divergence time for *Somanniathelphusa amoyensis* and *Somanniathelphusa taiwanensis* is approximately 0.80 Ma. The results indicate that the divergence time of the genus *Somanniathelphusa* of Parathelphusidae are consistent with the separation of Hainan Island from mainland China in the Beibu Gulf and the separation of Taiwan Province from Mainland China at Fujian Province, respectively. These data indicate that geologic events influenced speciation in the genus *Somanniathelphusa*.

## Supporting information

S1 Table Primers used in the present study. (DOCX)

S2 Table Brachyura species included in the present phylogenetic analysis. (DOCX)

S3 Table Brachyura species included in the present divergence time estimation. (DOCX)

S4 Table The base composition in different regions of mitochondrial genome of *Somanniathelphusa boyangensis*. (DOCX)
