# Supplementary material for: The complete mitochondrial genome of Somanniathelphusa boyangensis and phylogenetic analysis of Genus Somanniathelphusa (Crustacea: Decapoda: Parathelphusidae)
Source: PLoS One. 2018 Feb 13;13(2):e0192601. doi: 10.1371/journal.pone.0192601 (PMC5810993; doi:10.1371/journal.pone.0192601)
Supplement: S1 Table — (DOCX) [file pone.0192601.s001.docx]

**S1 Table. Primers used in the present study.**

| Primers | Sequences（5’-3’） | Target gene | References |
| --- | --- | --- | --- |
| 16SF | CCTAAMGAAYTTAGNGATRGCYGG | 16SRNA | Present study |
| 16SR | TAABCGYTGAACNAAMGAACC | 16sRNA | Present study |
| 12SF | CGGWTATACTACVTGRCNCAA | 12sRNA | Present study |
| 12SR | CTAGNCTGDTAVCTYTGRAGRGG | 12sRNA | Present study |
| COX1F | ATAHTAGRCGCCYCTDATAWAGC | COX1 | Present study |
| COX1R | GGAWAGYCCDAGYAAYGACDCGG | COX1 | Present study |
| ATP6F | GCCAACSCCSCTTWTCCNTATCC | ATP6 | Present study |
| ATP6R | GSTTGARTTATNGCGMCTGC | ATP6 | Present study |
| COX3F | GGGAGRGTACANTTYAAGG | COX3 | Present study |
| COX3R | GACTAMGTCRACAAAATGTYAG | COX3 | Present study |
| ND4F | CCARCCWCAGRAGYCTCNACWTG | ND4 | Present study |
| ND4R | GGAYGARGCDACYAGYCAGWGCG | ND4 | Present study |
| CYTBF | GCTACGTCCTCBCATGANGA | COB | Present study |
| CYTBR | GGTTRACCWCCGATTBAGG | COB | Present study |
| ND1F | CGARGTWGCRTCYCAYGGAYCCA | ND1 | Present study |
| ND1R | TACAWCCRGAYTCRGADAGGYCC | ND1 | Present study |
| SY-F1 | CTTTGTTCAACCATTCATAC | 16S-12S | Present study |
| SY-R1 | ATTAGATCAAGGTGCAGC | 16S-12S | Present study |
| SY-F2 | TAGGGTATCTAATCCTAG | 12S-COX1 | Present study |
| SY-R2 | AACAGTTCATCCTGTTCC | 12S-COX1 | Present study |
| SY-F3 | ACCAGTCCTTGCTGGTGC | COX1- ATP6 | Present study |
| SY-F4 | TACCTGTGTGTGATGGGG | COX1- ATP6 | Present study |
| SY-F5 | CCTTATCTTTACCTCTATG | ATP6-COX3 | Present study |
| SY-R5 | TGGGTTGATGCCTAAGGG | ATP6-COX3 | Present study |
| SY-F6 | GGGACAACATTTTTGTCAAC | COX3-ND4 | Present study |
| SY-R7 | GGTATTTTTTTGTAGTTTTGGC | COX3-ND4 | Present study |
| SY-F8 | AATAAAGGCAAAGACAATG | ND4-CYTB | Present study |
| SY-R8 | TCCTACTATTCAAGTGTGG | ND4-CYTB | Present study |
| SY-F9 | GTTCCTTTAATCCATTCATC | CYTB-ND1 | Present study |
| SY-R9 | TATGGCTGAATACGCAAG | CYTB-ND1 | Present study |
| SY-F10 | AACAAACTTAACATAGGAG | ND1-16S | Present study |
| SY-R10 | GTTTGTAACCTCGATGTTG | ND1-16S | Present study |
