# Supplementary material for: The complete mitochondrial genome of Somanniathelphusa boyangensis and phylogenetic analysis of Genus Somanniathelphusa (Crustacea: Decapoda: Parathelphusidae)
Source: PLoS One. 2018 Feb 13;13(2):e0192601. doi: 10.1371/journal.pone.0192601 (PMC5810993; doi:10.1371/journal.pone.0192601)
Supplement: S2 Table — (DOCX) [file pone.0192601.s002.docx]

**S2 Table.** **Brachyura species included in the present phylogenetic analysis.**

| Family | Species | Accession number |
| --- | --- | --- |
| Varunidae | *Helice latimera* | KU589291 |
| Varunidae | *Helice tientsinensis* | KR336555 |
| Varunidae | *Helicana wuana* | KX344898 |
| Varunidae | *Cyclograpsus granulosus* | LN624373 |
| Varunidae | *Eriocheir hepuensis* | FJ455506 |
| Varunidae | *Eriocheir japonica* | FJ455505 |
| Varunidae | *Eriocheir sinensis* | KP126617 |
| Varunidae | *Hemigrapsus sanguineus* | KX456205 |
| Sesarmidae | *Parasesarma tripectinis* | KU343209 |
| Sesarmidae | *Clistocoeloma sinense* | KU589292 |
| Sesarmidae | *Metopaulias depressus* | KX118277 |
| Sesarmidae | *Sesarmops sinensis* | KR336554 |
| Sesarmidae | *Sesarma neglectum* | KX156954 |
| Grapsidae | *Grapsus tenuicrustatus* | KT878721 |
| Grapsidae | *Pachygrapsus crassipes* | KC878511 |
| Ocypodidae | *Ocypode cordimanus* | KT896743 |
| Ocypodidae | *Ocypode ceratophthalmus* | LN611669 |
| Macrophthalmidae | *Macrophthalmus japonicus* | KU343211 |
| Mictyridae | *Mictyris longicarpus* | LN611670 |
| Dotillidae | *Ilyoplax deschampsi* | JF909979 |
| Xenograpsidae | Xenograpsus testudinatus | EU727203 |
| Portunidae | *Scylla olivacea* | FJ827760 |
| Portunidae | *Scylla serrata* | HM590866 |
| Portunidae | *Scylla paramamosain* | JX457150 |
| Portunidae | *Scylla tranquebarica* | FJ827759 |
| Portunidae | *Charybdis feriata* | KF386147 |
| Portunidae | *Charybdis japonica* | FJ460517 |
| Portunidae | *Thalamita crenata* | LK391945 |
| Portunidae | *Callinectes sapidus* | AY363392 |
| Portunidae | *Portunus sanguinolentus* | KT438509 |
| Portunidae | *Portunus pelagicus* | KR153996 |
| Portunidae | *Portunus trituberculatus* | AB093006 |
| Potamidae | *Potamiscus motuoensis* | KY285013 |
| Potamidae | *Sinopotamon yangtsekiense* | JF909980 |
| Potamidae | *Geothelphusa dehaani* | AB187570 |
| Potamidae | *Sinopotamon xiushuiense* | KU042041 |
| Potamidae | *Huananpotamon lichuanse* | KX639824 |
| Parathelphusidae | *Somanniathelphusa boyangensis* | KU042042 |
| Bythograeidae | *Austinograea alayseae* | KC851803 |
| Bythograeidae | *Austinograea rodriguezensis* | JQ035658 |
| Bythograeidae | *Gandalfus puia* | KR002727 |
| Bythograeidae | *Gandalfus yunohana* | EU647222 |
| Bythograeidae | *Segonzacia mesatlantica* | KY541839 |
| Majidae | *Maja crispata* | KY650651 |
| Majidae | *Maja squinado* | KY650652 |
| Matutidae | *Ashtoret lunaris* | LK391941 |
| Leucosiidae | *Pyrhila pisum* | KU343210 |
| Mithracidae | *Damithrax spinosissimus* | KM405516 |
| Xanthidae | *Leptodius sanguineus* | KT896744 |
| Menippidae | *Myomenippe fornasinii* | LK391943 |
| Geryonidae | *Chaceon granulatus* | AB769383 |
| Eriphiidae | *Pseudocarcinus gigas* | AY562127 |
| Raninidae | *Ranina ranina* | AB752308 |
| Raninidae | *Lyreidus brevifrons* | KM983394 |
| Raninidae | *Umalia orientalis* | KM365084 |
| Homolidae | *Homologenus malayensis* | KJ612407 |
| Homolidae | *Moloha majora* | KT182069 |
| Dynomenidae | *Dynomene pilumnoides* | KT182070 |
| Palinuridae | *Panulirus ornatus* | GQ223286 |
