# Supplementary material for: The complete mitochondrial genome of Somanniathelphusa boyangensis and phylogenetic analysis of Genus Somanniathelphusa (Crustacea: Decapoda: Parathelphusidae)
Source: PLoS One. 2018 Feb 13;13(2):e0192601. doi: 10.1371/journal.pone.0192601 (PMC5810993; doi:10.1371/journal.pone.0192601)
Supplement: S3 Table — (DOCX) [file pone.0192601.s003.docx]

**S2 Table.** **Brachyura species included in the present divergence time estimation.**

| Family | Species | Accession number |
| --- | --- | --- |
| Parathelphusidae | *Somanniathelphusa qiongshanensis* | AB265248 |
| Parathelphusidae | *Somanniathelphusa zhapoensis* | AB265246 |
| Parathelphusidae | *Somanniathelphusa amoyensis* | AB265242 |
| Parathelphusidae | *Somanniathelphusa taiwanensis TW-C1* | AB265239 |
| Parathelphusidae | *Somanniathelphusa taiwanensis TW-C1-1* | AB265240 |
| Parathelphusidae | *Somanniathelphusa taiwanensis TW-C3* | AB265241 |
| Parathelphusidae | *Somanniathelphusa zanklon ZL-C1* | AB265244 |
| Parathelphusidae | *Somanniathelphusa zanklon ZL-C2* | AB265245 |
| Parathelphusidae | *Somanniathelphusa zanklon ZL-C3* | AB265247 |
| Homolidae | *Moloha majora* | KT182069 |
| Homolidae | *Homola orientalis* | KT182071 |
| Homolidae | *Homologenus malayensis* | KJ612407 |
| Raninidae | *Umalia orientalis* | KM365084 |
| Raninidae | *Lyreidus brevifrons* | KM983394 |
| Raninidae | *Ranina ranina* | AB752308 |
| Portunidae | *Scylla olivacea* | FJ827760 |
| Portunidae | *Scylla paramamosain* | JX457150 |
| Portunidae | *Scylla tranquebarica* | FJ827759 |
| Portunidae | *Scylla serrata* | HM590866 |
| Portunidae | *Charybdis feriata* | KF386147 |
| Portunidae | *Charybdis japonica* | FJ460517 |
| Portunidae | *Portunus pelagicus* | KR153996 |
| Portunidae | *Callinectes sapidus* | AY363392 |
