# Supplementary material for: The complete mitochondrial genome of Somanniathelphusa boyangensis and phylogenetic analysis of Genus Somanniathelphusa (Crustacea: Decapoda: Parathelphusidae)
Source: PLoS One. 2018 Feb 13;13(2):e0192601. doi: 10.1371/journal.pone.0192601 (PMC5810993; doi:10.1371/journal.pone.0192601)
Supplement: S4 Table — (DOCX) [file pone.0192601.s004.docx]

**S4 Table. The base composition in different regions of mitochondrial genome of *Somanniathelphusa boyangensis* (the genes which are encoded by L-strand are converted to complementary strand sequences).**

| Region | Base composition (%) | | | | A+T content(%) |
| --- | --- | --- | --- | --- | --- |
|  | A | G | T | C |  |
| *Protein-coding gene* |  |  |  |  |  |
| *COX1* | 28.2 | 15.6 | 37.1 | 19.1 | 65.3 |
| *COX2* | 32.3 | 11.4 | 36.4 | 19.9 | 68.7 |
| *ATP8* | 30.8 | 4.4 | 45.9 | 18.9 | 76.7 |
| *ATP6* | 30.1 | 9.5 | 38.4 | 22.0 | 68.5 |
| *COX3* | 27.6 | 13.7 | 36.2 | 22.5 | 63.8 |
| *ND3* | 29.3 | 11.0 | 42.3 | 17.4 | 71.6 |
| *ND4* | 29.6 | 19.0 | 43.0 | 8.4 | 72.6 |
| *ND4L* | 26.4 | 23.4 | 44.9 | 5.3 | 71.3 |
| *ND6* | 28.3 | 8.0 | 44.7 | 19.0 | 73.0 |
| *Cyt b* | 28.5 | 11.6 | 40.9 | 18.9 | 69.4 |
| *ND5* | 29.6 | 20.0 | 41.6 | 8.8 | 71.2 |
| *ND1* | 27.2 | 19.0 | 45.7 | 8.1 | 72.9 |
| *ND2* | 27.2 | 8.6 | 43.8 | 20.4 | 71.0 |
|  |  |  |  |  |  |
| *tRNA gene* |  |  |  |  |  |
| *tRNA^Leu（UUR）^* | 38.1 | 17.5 | 31.7 | 12.7 | 69.8 |
| *tRNA^Lys^* | 33.8 | 16.9 | 38.5 | 10.8 | 72.3 |
| *tRNA^Asp^* | 33.3 | 12.7 | 39.7 | 14.3 | 73.0 |
| *tRNA^Gly^* | 41.9 | 9.7 | 33.9 | 14.5 | 75.8 |
| *tRNA^Ala^* | 32.3 | 14.5 | 41.9 | 11.3 | 74.2 |
| *tRNA^Ser（AGN）^* | 33.3 | 13.6 | 33.3 | 19.7 | 66.6 |
| *tRNA^Glu^* | 37.5 | 7.8 | 46.9 | 7.8 | 84.4 |
| *tRNA^His^* | 34.3 | 4.5 | 44.8 | 16.4 | 79.1 |
| *tRNA^Thr^* | 38.1 | 12.7 | 38.1 | 11.1 | 76.1 |
| *tRNA^Ser(UCN)^* | 44.8 | 10.4 | 37.3 | 7.5 | 82.1 |
| *tRNA^Gln^* | 32.4 | 22.1 | 41.2 | 4.4 | 73.6 |
| *tRNA^Cys^* | 41.5 | 16.9 | 33.8 | 7.7 | 75.3 |
| *tRNA^Arg^* | 33.3 | 13.6 | 36.4 | 16.7 | 69.7 |
| *tRNA^Asn^* | 36.6 | 12.7 | 35.2 | 15.5 | 71.8 |
| *tRNA^Phe^* | 50.8 | 11.1 | 33.3 | 4.8 | 84.1 |
| *tRNA^Pro^* | 37.9 | 18.2 | 37.9 | 6.1 | 75.8 |
| *tRNA^Leu(CUN)^* | 44.6 | 13.8 | 35.4 | 6.2 | 80.0 |
| *tRNA^Val^* | 32.9 | 13.7 | 41.1 | 12.3 | 74.0 |
| *tRNA^Ile^* | 36.9 | 15.4 | 33.8 | 13.8 | 70.7 |
| *tRNA^Met^* | 36.4 | 15.2 | 30.3 | 18.2 | 66.7 |
| *tRNA^Trp^* | 37.9 | 9.1 | 37.9 | 15.2 | 75.8 |
| *tRNA^Tyr^* | 32.3 | 23.1 | 35.4 | 9.2 | 67.7 |
|  |  |  |  |  |  |
| *rRNA gene* |  |  |  |  |  |
| *16S rRNA* | 42.1 | 16.3 | 34.9 | 6.7 | 77.0 |
| *12S rRNA* | 41.6 | 15.9 | 35.6 | 6.8 | 77.2 |
| *Putative control region* | 43.1 | 1.6 | 44.7 | 10.6 | 87.8 |
| *Overall of protein-coding genes* | 28.8 | 14.8 | 41.1 | 15.4 | 69.9 |
| *Overall of tRNA genes* | 37.3 | 13.9 | 37.2 | 11.7 | 74.5 |
| *Overall of rRNA genes* | 41.9 | 16.2 | 35.2 | 6.8 | 77.1 |
| *Overall of the genome* | 35.1 | 9.2 | 37.2 | 18.4 | 72.3 |
